# Comparative Phytochemical Profiles and Antioxidant Enzyme Activity Analyses of the Southern Highbush Blueberry (*Vaccinium corymbosum*) at Different Developmental Stages

**DOI:** 10.3390/molecules23092209

**Published:** 2018-08-31

**Authors:** Yueting Sun, Min Li, Sangeeta Mitra, Rizwan Hafiz Muhammad, Biswojit Debnath, Xiaocao Lu, Hongxiang Jian, Dongliang Qiu

**Affiliations:** 1 College of Horticulture, Fujian Agriculture and Forestry University, Fuzhou 350002, Fujian, China; yuetingsun@126.com (Y.S.); liminzyl@sina.com (M.L.); sangeeta.dae@hotmail.com (S.M.); chrizwan51@gmail.com (R.H.M.); biswo26765@yahoo.com (B.D.); xc531599541@126.com (X.L.); 2 Guyue Mountain Farmer, Xiamen 361000, Fujian, China; hxjian2002@163.com

**Keywords:** phenolics, flavonoids, anthocyanins, antioxidant activity, blueberry

## Abstract

In this study, the fruit quality, anthocyanin content and antioxidant enzyme activities of skin and pulp of southern blueberries (*Vaccinium corymbosum*) from China y at five developmental stages (green, pink, red, purple and blue) were investigated and anthocyanins were characterized and quantified by HPLC during the considered developmental stages. The results indicatated that the contents of phenolic, flavonoids and anthocyanin as well as antioxidant enzyme activities varied depending on the developmental stages. The correlation values between total phenolic content (TPC), total flavonoids content (TFC) and total anthocyanin content (TAC) were significant. The highest activity of peroxidase (POD) and catalase (CAT) was found in red fruit, and the variety of monomeric anthocyanin increased gradually, skin from blue fruit possessed higher TAC. However, the highest activity of polyphenol oxidase (PPO) and superoxide dismutase (SOD) were detected in green and blue fruit, respectively. In the present work, the differences regarding phytochemical profiles and antioxidant enzyme activities were mainly correlated with developmental stages of fruit.

## 1. Introduction

Growing amount of studies on natural antioxidants have been conducted in recent years due to high content of bioactive components [1], and people’s concern about the use of synthetic antioxidants. Nowadays, synthetic antioxidants are widely used in pharmaceuticals, cosmetics and food products, but their long-term use is accompanied by toxic and other side effects [2].

Blueberry (*Vaccinium corymbosum*) belongs to the Ericaceous Vaccinium group of deciduous shrub plants. Antioxidant chemicals in fruits include not only the substances such as folate and phenolics, and flavonoids, especially anthocyanins, characterized by high antioxidant activity, but also enzymes such as SOD, POD, PPO and CAT, etc., hence its worldwide popularity due to its high nutritional value [3,4]. More recent studies have illustrated that blueberry could prevent heart diseases, inhibit cancer cell proliferation [5], inflammation [6], obesity and diabetes [7]. They contain a lot of phenolic compounds such as chlorogenic acid, quercetin, catechin, epicatechin and vitamin C [8]. Anthocyanins are water-soluble pigments and natural colorants, being one of the reasons that fruita, flowers and vegetables show colors. Anthocyanins are used to color jams, ice creams and other foods. They are also the most effective natural antioxidants and are shown to have significant anti-aging, anti-cancer and immunoprotective effects [4,9,10]. The antioxidant enzyme activities in blueberries are complex processes that are not completely understood. The influence of factors such as genes on antioxidant enzymes is very profound [11]. The main antioxidant enzymes in crops like blueberries, blackberries (*Rubus fruticosus*), deerberries (*Vaccinium stamineum* L.), and strawberries (*Fragaria ananassa* et al.) are SOD, POD, PPO and CAT [12,13].

The composition of the phytochemical profiles in blueberries varies widely, according to the cultivar, season, location, harvesting, storage and the degree of maturity at harvest [8,14,15]. Exploring the changes of phytochemical profiles and antioxidant enzyme activities at different fruit growth periods has great importance to further study the mechanism of action of blueberry. Recently, there are many studies about the antioxidant potential of blueberry fruit [3,4,16], but these studies have focused primarily on the phytochemical profiles and antioxidant activities. However, it is not clear how the developmental stages of fruit influence the phytochemical compound and antioxidant activities of blueberry. Therefore, with an aim to explore the variations of phytochemical profiles and antioxidant enzyme activities of different developmental stages of fruit to evaluate their antioxidant activity, this study focus on: (1) to evaluate the soluble sugar, titratable acidity, vitamin C, total phenolic, flavonoids, anthocyanin content of blueberry in different developmental stages (green, pink, red, purple, blue), skin and pulp; (2) qualitative analysis of anthocyanin in different maturity fruit; (3) to determine the antioxidant enzyme activities in terms of SOD, POD, PPO and CAT; (4) correlation analysis between blueberry chemical composition and enzyme activities. 

## 2. Results and Discussion

### 2.1. Growth Data and Water Content of Different Developmental Stages of Fruit

The growth data of blueberry fruit at different developmental stages, are shown in Table 1. The transverse diameter, vertical diameter and fruit weight were all increased from the green fruit stage to blue fruit stage. The fresh weight and dry weight were all increased, too. Fruit weight was increased from 0.43 g to 1.11 g during fruit ripening. However, water content was stable during the different developmental stages. 

### 2.2. Influence of Different Developmental Stages of Fruit on Soluble Sugars (SS) and Titratable Acidity (TA)

**Figure 1:**
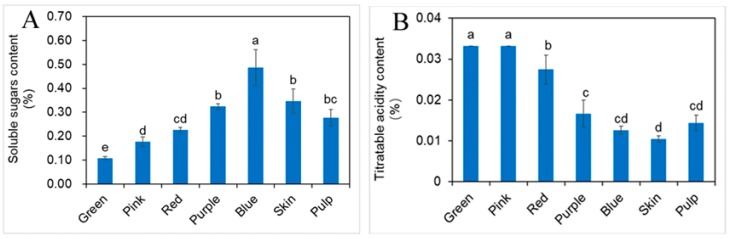
Changes of the contents of soluble sugar (**A**) and titratable acidity (**B**) for blueberry at different developmental stages of fruit, skin and pulp (mean ± SD, *n* = 3). Bars with different letters differ significantly (*p* < 0.05).

The contents of soluble sugar and titratable acidity in blueberry fruit (Figure 1) showed opposite patterns of change during development. SS contents of fruits (ranging from 0.11 to 0.49%) significantly increased in a straight line and TA contents (ranged from 0.03 to 0.01%) are in a dynamic downward trend. In addition, SS contents of skin and pulp are about 0.1 percent lower than in fruit. In agreement with our research, some previous reports also revealed that the soluble sugars varied with fruit maturity, depending on the species, varieties, cultivars, cultivation pattern and harvest time [17,18]. These results indicated that sweet and sour taste of blueberry is related directly to the change of SS and TA during fruit development [19]. 

### 2.3. Effects of Different Developmental Stages of Fruit on Phenolic, Flavonoids and vit. C Contents

**Figure 2:**
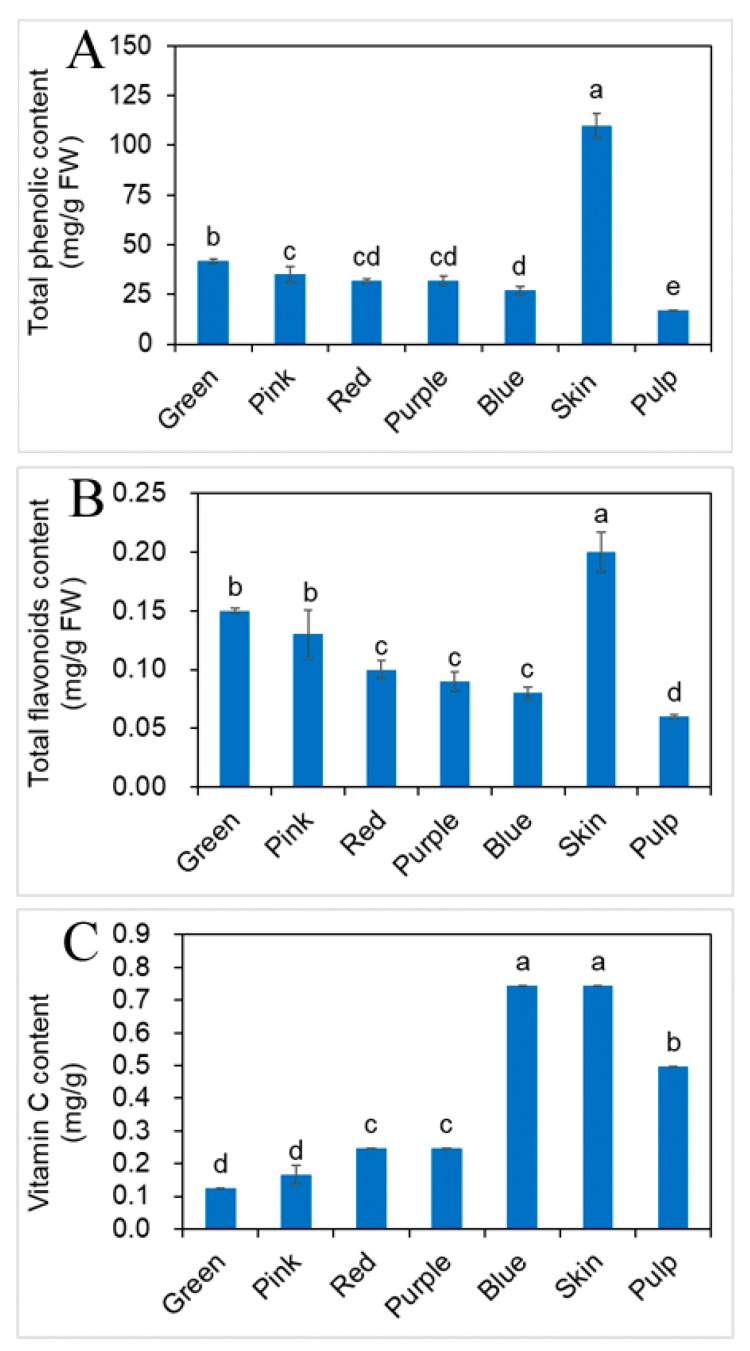
Changes of the contents of phytochemical profiles for blueberry at different developmental stages of fruit, skin and pulp. (**A**) Total phenolic content; (**B**) total flavonoids content; (**C**) vitamin C content (mean ± SD, *n* = 3). Bars with different letters differ significantly (*p* < 0.05).

The total phenolic content (TPC), total flavonoids content (TFC) and vitamin C content (vit. C) of the different developmental stages of fruit, skin and pulp of the blueberry were determined (Figure 2). 

In the different developmental stages of fruit, TPC was decreased as the fruit grows. TPC in green fruit was highest (42.35 mg/g FW), and blue fruit was lowest (26.59 mg/g FW), the result is consistent with Eichholz et al. [20]. TPC of skin and pulp, were 110.48, 17.32 mg/g FW, respectively. TPC of skin is four times than in the blue fruit, and six times the level in the pulp. These contents were significantly different (*p* < 0.05). TPC tended to be highest in the skin, followed by the fruit and the pulp. TPC of the skin was significantly higher than the fruit and pulp contents (*p* < 0.05), while TPC of the pulp was not significantly lower than the fruit contents (*p* > 0.05). Castrejón et al. [21] examined TPC of Reka, Puru, Bluecrop and Berkeley at different developmental stages of fruit, TPC decreased and ranged in average from 60.76 (green fruit) to 33 (blue fruit) mg/g DM. Kalt et al. [22] examined TPC of Bergitta, Bluegold and Nelson at different developmental stages of fruit, TPC decreased and ranged in average from 21.36 (green fruit) to 15.13 (blue fruit) mg/g DM. In agreement with previous reports, the values in the present study ranged in average from 42 (green fruit) to 27 (blue fruit) mg/g FW. Differences of the present results in comparison to the one reported may arise due to different cultivation and climate conditions or using different extraction solvents in sample extraction.

TFC had same trend as TPC at different developmental stages of fruit. The green fruit had the highest TFC at 0.15 mg/g FW, and blue fruit had lowest TFC at 0.08 mg/g FW. TFC of skin and pulp, were 0.20, 0.06 mg/g FW, respectively. TFC of skin was 2.5 times as high as the blue fruit, and three times higher than the pulp. TFC of skin, pulp and blue fruit were significantly different (*p* < 0.05), but at the different developmental stages of fruit, all (green, pink, red, purple and blue fruit) had similar flavonoid contents (*p* > 0.05). The fruits from different ripening stages are one of reasons in different composition and content of both flavonoids and titratable acidity [23]. 

The vit. C content of the skin was highest in blue fruit which possessed the highest content (0.75 mg/g), where the pulp content was 0.50 mg/g. The vit. C levels of green, pink, red and purple fruit, were 0.12, 0.17, 0.25 and 0.25 mg/g, respectively. This trend was seen many fruits such as strawberries, mulberries (*Morus* spp.) and pomegranates (*Punica granatum* L.) during their developmental stages [17,24]. The vit. C levels of skin, pulp and blue fruit were not significantly different (*p* > 0.05), but these values were significantly different when compared to that content of green, pink, red and purple fruit (*p* < 0.05). The TPC, TFC, vit. C contents of skin were much higher than in other tissues, which may indicate that skin has the higher antioxidant performance. The phytochemical profiles of pulp were less than in the fruit at the same developmental stage, which was consistent with the results.

Some studies have showed that flavonoid biosynthesis is tightly associated with the developmental stages of fruit. Bilberry (*Vaccinium myrtillus*) shows a coordinated expression of flavonoid biosynthetic genes in relation to the accumulation of anthocyanins, proanthocyanidins, and flavonols during fruit maturation [25]. It has been demonstrated that flavonoid biosynthesis has one distinct key flavonoid enzyme activity peak during the development stages of the fruit, such as strawberries, grapes (*Vitis vinifera*) and other berry fruits [21,26].

### 2.4. Total Anthocyanin Content (TAC) and Qualitative Identification by HPLC

**Figure 3:**
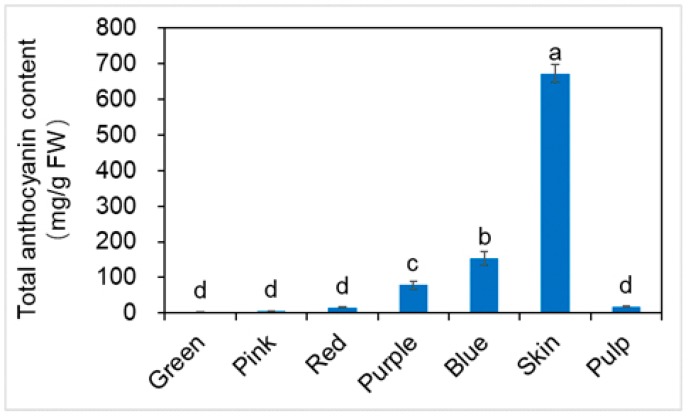
Changes of the contents of total anthocyanin for blueberry different developmental stages of fruit, skin and pulp (mean ± SD, *n* = 3). Bars with different letters differ significantly (*p* < 0.05).

TAC of blueberry fruit was shown in Figure 3. TAC of the different developmental stages of fruit was 3.43, 5.46, 16.14, 78.90, and 153.39 mg/g FW, green, pink, red, purple and blue fruit, respectively. The maturation stages contained the highest level of anthocyanins among developmental stages of fruit [27]. TAC of skin and pulp, were 672.12, 18.09 mg/g FW, respectively. TAC of skin was four times as high as the blue fruit, and 37 times the level of the pulp. TAC of skin, pulp and blue fruit were significantly different (*p* < 0.05). TAC tended to be highest in the skin. 

These results indicated that TFC, TPC and TAC of blueberry are mainly present in the skin. There were no significant differences in TAC of green, pink, and red fruit (*p* > 0.05). There are studies that have shown that the transition from green fruit to blue fruit is a result of the accumulation of anthocyanins [28].

**Figure 4:**
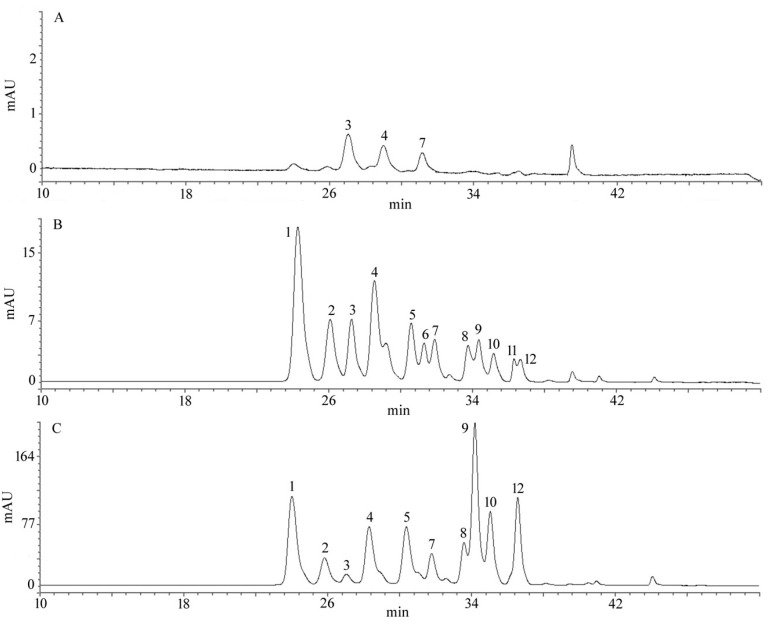
HPLC results of anthocyanins in different developmental stages of blueberry fruit. (**A**) green fruit; (**B**) red fruit; (**C**) blue fruit. Using HPLC at 520 nm (mean ± SD, *n* = 3).

For HPLC analysis, we only selected the three most representative periods (green fruit, red fruit and blue fruit) to determine the phytochemicals present. According to the standard samples and references, a total of 12 anthocyanin compounds of the green fruit, red fruit and blue fruit were identified in the HPLC chromatograms. As shown in Table 2 and Figure 4, good separation can be achieved between each anthocyanin component. They belonged to five main groups of anthocyanins including delphinidin, cyanidin, peonidin, petunidin and malvidin. The individuals and content of anthocyanin are increasing in developmental stages. The major components of anthocyanin are different at developmental stages of fruit. The main anthocyanin at the green-fruit stage was cyanidin, where the main anthocyanin at the red-fruit stage was delphinidin. However, the main anthocyanin found at the blue-fruit stage was malvidin. The acylated anthocyanin was presented in red and blue fruit, this finding was consistent with the results of other authors [8,29]. Delphinidin was the most major anthocyanin in blueberries, malvidin was followed by delphinidin. In addition, the anthocyanin content of blue fruit was higher than the green, pink, red and purple fruit. The development of the anthocyanin content had mainly manifested that the fruit presents a change from green to blue. These findings indicate that the changes of blueberry colour may be greatly affected by the anthocyanin compounds, during developmental stages [30].

### 2.5. Influence of Different Developmental Stages of Fruit on Antioxidant Enzyme Activity

**Figure 5:**
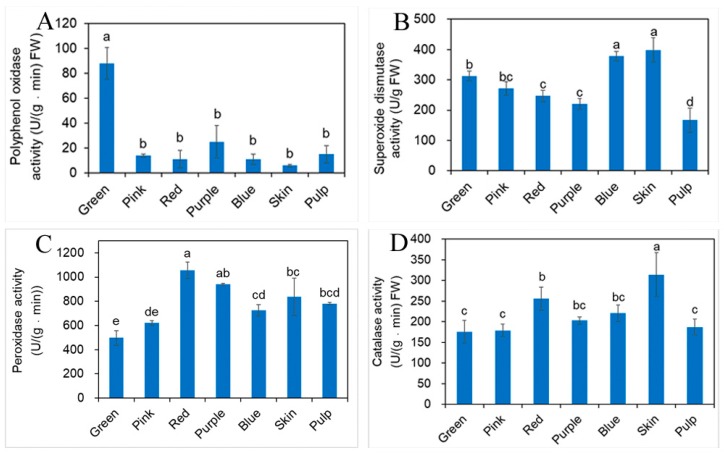
Changes of the contents of antioxidant enzyme activity for blueberry different developmental stages of fruit, skin and pulp. (**A**) Polyphenol oxidase activity; (**B**) superoxide dismutase activity; (**C**) peroxidase activity; (**D**) catalase activity (mean ± SD, *n* = 3). Bars with different letters in common differ significantly (*p* < 0.05).

The antioxidant enzyme activities as measured are presented in Figure 5, obvious differences in the antioxidant enzyme activities were observed among different developmental stages of fruit and tissues. The highest activity of PPO, SOD, POD, or CAT was detected in the green, blue, red, and red fruit stages, respectively. In addition, higher activities of SOD, CAT were found in skin. Generally, there was no significant difference in the content of the skin and the fruit at the blue stage. In the skin, pulp and blue fruit, generally SOD, POD, and CAT were the highest in the skin, followed by the blue fruit, and the pulp content was the lowest, while the opposite was found in the PPO. It may be that during the separation process through the air, the enzyme activity was increased, leading to the highest activity of PPO in pulp. The antioxidant enzyme activities in fruit are mainly influenced by species, environmental conditions, fruit maturation, variability over the years, harvest season and other factors [31,32]. In this study, the blueberries were used in that same species, growth conditions, effects of climate, soil condition and year of harvest, so the enzyme activities are controlled in response to different developmental cues.

The correlation among TPC, TFC, TAC, vit. C, SOD, POD, PPO and CAT of blueberry is shown in Table 3. TFC, TAC at different fruit stage of southern blueberry represented a significantly positive correlation with TPC showing the very high correlation coefficient (0.91, 0.93; 0.8 < r < 1; *p* < 0.01) in our study, respectively. This result was supported by Moyer et al. that high correlations between TAC and TPC with different antioxidant screening methods like blueberries, rubus (*Rosaceae*) and ribes were observed [33]. Kalt et al. reported that the increase of TAC and decrease of TPC at developmental stages of fruit. They pointed out that there is a shift of total phenolics toward anthocyanin synthesis, and the content of other phenolic components decline overall at developmental stages of fruit [34]. TAC had a moderate positive correlation with vit. C calculated for different fruit stages (0.70; 0.5 < r < 0.8; *p* < 0.01). SOD and CAT showed a moderate positive correlation with TPC, TFC, TAC and vit. C. PPO with POD showed moderate correlation. CAT also showed moderate correlation with SOD, PPO. No correlation between POD, SOD with TPC, TFC and TAC was found at different fruit stages. POD and PPO have been implicated in cellular protection and disease resistance to oxidize phenolics substances into quinones in the process of tissue aging [35]. POD is an enzyme with important roles in enhancing antioxidant capacity [36], which promotes the oxidation of phenolics and the formation of ethylene and the increase of respiratory strength. . Total phenolics content was related to POD or PPO activities in all probability at different developmental stages of blueberry fruit. POD and CAT increase relatively with fruit ripening, probably because the increase of free radical production and defensive response produced by the fruit itself, and these enzymes neutralized free radicals in fruit. SOD activity is quite different from the changes of other enzymes. It is an important antioxidant enzyme that eliminate oxygen free radicals in cells. The relative decrease of these enzyme activities during developmental stages may be due to the conversion and decomposition of enzymes to inactivation. The metabolism and antioxidant activity are determined by the enzyme activity and phytochemical profiles through complex interactions at the different developmental stages of blueberry fruit, rather than simply caused by the change of a certain material.

## 3. Materials and Methods

### 3.1. Plant Materials

**Figure 6:**
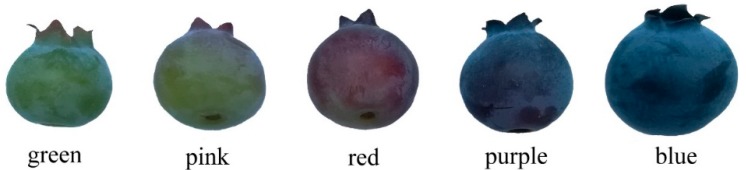
Developmental stages of blueberry fruit used for phytochemical profiles and antioxidant activity analyses.

The southern highbush cultivar ‘FLS03’ fruit samples were directly collected in April-May, 2018 from the producer at the GuYue Mountain Farmer (coordinates 117°59′55″ S and 24°37′11″ N), Jimei District, Xiamen, Fujian, China. Blueberries at different developmental stages of fruit (green, pink, red, purple and blue) were showed in Figure 6. Collected fruit samples were carried back to the lab, then skin and pulp of blue fruit samples were separated manually and other stages of fruit frozen immediately in liquid nitrogen, and stored at −80 °C until use. 

### 3.2. Instruments and Chemicals

The ultrasonic cleaner was obtained from Kunshan Ultrasonic Instrument Co. (Kunshan, China). The high speed bench centrifuge was from Xima Centrifuge Co., Ltd. (Yangzhou, China). The high performance liquid chromatography (HPLC) system was purchased from Shanghai Wufeng Scientific Instruments Co., Ltd. (Shanghai, China). UV-VIS spectrophotometer was purchased from Shanghai Yuanxi Instrument Co. Ltd. (Shanghai, China). Only chemicals of analytical grade were used, concentrate hydrochloric acid, methanol, aluminum nitrate, sodium hydroxide, anthrone, ethyl acetate, sucrose, concentrated sulfuric acid, potassium acid phthalate, phenolphthalein, ethanol, sodium carbonate, copper sulfate pentahydrate, sodium nitrite, activated carbon, ascorbic acid, gallic acid, rutin, Folin-Ciocalteau reagent (FC), catechol, disodium phosphate dodecahydrate, sodium dihydrogen phosphate, L-methionine (Met), nitroblue tetrazolium (NBT), ethylenediaminetetraacetic acid disodium salt, riboflavin, 2-methoxyphenol, 30% hydrogen peroxide. Standards of anthocyanin (delphinidin-3-glucoside, cyanidin-3-glucoside, petunidin-3-glucoside, peonidin-3-glucoside, malvidin-3-glucoside), formic acid, were obtained from Fuzhou Nanjiang Biotechnology (Fuzhou, China).

### 3.3. Growth Data and Water Content

Transverse diameter and vertical diameter were measured on five fresh fruits from each stage. Then they were dried separately to a constant weight. The dry weight and water content were calculated using the equations listed below. Dry weight (%) = (Dry weight of fruit/fruit before drying) × 100%. Water content (%) = ((fruit weight before drying − fruit weight after drying)/fruit weight before drying) × 100%.

### 3.4. Soluble Sugars (SS)

The soluble sugars content was quantified by the method described by Zhu et al. [37]. A total of 0.2 g of each sample was ground in liquid nitrogen and the powdery sample was homogenized in 10 mL water, then a suitable amount of activated carbon was added and the sample placed in a boiling water bath for 30 min (repeated twice). After cooling, the samples were centrifuged at 8000 RPM for 5 min, then the supernatant was collected and made up to a final volume of 25 mL with distilled water and used for further analysis. Supernatant (0.5 mL) was added into a 10 mL tube containing 2 mL of distilled water, 0.5 mL anthrone and ethyl acetate was added into the mixture. Then, 5 mL concentrated sulfuric acid was added slowly into the mixture. The mixture was boiled in the water bath for 1 min, and after cooling to room temperature, its absorbance was measured against a blank at 630 nm using a UV-VIS spectrophotometer. The equation obtained for the calibration curve of sucrose in the range of 20–100 µg/mL was y = 0.006x − 0.0259 (R^2^ = 0.9913).

### 3.5. Titratable Acidity (TA)

A total of 0.2 g of each sample were ground in liquid nitrogen, and 10 mL water was added to the powdery sample, then a suitable amount of activated carbon was added to completely remove the color and the sample was boiled in a water bath for 30 min at 80 °C. After cooling, the samples were centrifuged at 8000 RPM for 5 min, then the supernatant was collected in another tube and the procedure repeated. Again, the supernatant was removed and made up to a final volume of 25 mL with distilled water for further analysis. Supernatant (10 mL) was placed in a 50 mL conical flask and 3 to 5 drops of phenolphthalein indicator were added. Then the mixture was titrated with 0.1 N NaOH solutions until a pink color which did not fade within 30 sec appeared. The initial and final volume was recorded for calculation of TA [38].

### 3.6. Total Phenolic Content (TPC)

For estimation of total phenolics, flavonoids, anthocyanin samples of each development stage of fruit were extracted according to the method described by Pastrana et al. [39]. Briefly, a 0.5 g powdery sample was homogenized in 5 mL of 2% HCl-methanol solution and further mixed. After ultrasonic irradiation for 60 min, the extracts were centrifuged at 4 °C and 12000 RPM for 10 min. Then the supernatant was filtered and collected for further analysis. The total phenolic content was measured by the FC method with minor modification [40,41]. FC-A (5 mL) was added to the extract sample (1 mL) and this mixture was left standing for 10 min at 25 °C before the addition of FC-B reagent (0.5 mL). The solution was then kept for 30 min at 25 °C before measurement at 500 nm. Total phenolic content was expressed as gallic acid standard equivalents on a fresh weight basis (mg/g FW). The equation obtained for the calibration curve of gallic acid in the range of 10–50 µg/mL was y = 0.0006x − 0.0009 (R^2^ = 0.9953).

### 3.7. Total Flavonoids Content (TFC)

TFC was determined according to Wolfe et al. [42]. Sample solution (0.4 mL) was added to a 10 mL test tube containing water (2 mL). After that 5% sodium nitrite solution (0.12 mL) was added and incubated for 5 min at room temperature, then 10% aluminum nitrate solution (0.24 mL) was added to the mixture. After 6 min, 1 mol/L sodium hydroxide (0.8 mL) was added and the mixture was diluted with water (0.44 mL). The absorbance was measured at 420 nm. The equation obtained for the calibration curve of rutin standard solution in the range of 80–400 µg/mL was y = 3.3454x − 0.0039 (R^2^ = 0.9950) and result was expressed as mg of rutin (rutin equivalents) per g of fresh weight (FW).

### 3.8. Total Anthocyanin Content (TAC)

TAC in the sample was determined according to Fuleki et al. [43], with 2% HCL-methanol solution as the control. The absorbance was measured at 520 nm. TAC was calculated as follows: TAC (mg·g^−1^ FW) = A_520_ × M_W_ × a × 1000/(ε × 1), where A_520_ is absorbance; M_W_ is molecular weight for cyanidin-3-glucoside = 449.2; a is dilute multiple; ε is the molar absorptivity of cyanidin-3-glucoside = 26,900; and 1 is 1 cm colorimetric utensil. TAC was expressed as mg of cyanidin 3-glucoside equivalents per g of fresh weight.

### 3.9. Vitamin C content (vit. C)

The vitamin C content was quantified by the oxalic acid titration method described by Wang et al. [38]. The vitamin C content expressed as mg of ascorbic acid (ascorbic acid equivalents) per g of fresh weight.

### 3.10. Anthocyanin Qualitative Identification by HPLC

HPLC analysis for anthocyanin was analyzed by following the procedures described by Grace et al. [29]. The analysis of anthocyanins was performed on an LC100 HPLC series system equipped with a Waters C18 column (250 mm × 4.6 mm × 5 µm). The detection wavelength was 520 nm, column temperature was 20 °C, and the injection volume was 20 μL. The mobile phase consisted of 5% formic acid water solution (phase A) (*v*/*v*) and 100% methanol (phase B) at a flow rate of 1.0 mL/min. Gradient elution was performed as follows: a gradient of 10–15% B for 5 min; 15–20% B for 5 min; a gradient of 20–25% B for 5 min; 25–30% B for 5 min; and then a gradient of 30–60% B for 20 min; a final wash with 60–10% for 2 min. The operation time was 60 min. Four concentrations of cyanidin-3-glucoside were prepared at 1.25, 2.5, 5 and 10 μg/mL as an external standard. The equation curve of cyanidin-3-glucoside standard solution was y = 0.1192x + 0.4414, (R^2^ = 0.9974).

### 3.11. Polyphenol Oxidase Activity (PPO)

PPO activity was determined according to Zhu et al. with a minor modification [37]. Powdery sample (0.2 g) was added into precooled 0.05 mol/L pH 7.0 PBS solution (3 mL). Then the sample mixture was centrifuged at 4 °C, 12,000 RPM for 20 min and the supernatant was collected and used as enzyme solution (all operations were done on the ice). Catechol solution (0.05 mL, 0.1 mol/L) was taken in a test tube, then pH 7.0 PBS solution (1.0 mL, 0.05 mol/L) was added in it. After that enzyme solution (0.5 mL, control is 0.5 mL distilled water) was added into the test tube. It was shaken well for mixing and then kept it in a water bath at 37 °C for 10 min. Absorbance was taken at 410 nm (3 min). The change of absorbance of 0.01 per min was taken as 1 enzyme activity unit (U).

### 3.12. Superoxide Dismutase Activity (SOD)

SOD activity was determined following the method of Rao et al. [44] with a minor modification. Powdery sample (0.3 g) was added into precooled 0.05 mol/L pH 7.8 phosphate buffer (3 mL). Then the sample mixture was centrifuged at 4 °C, 12,000 RPM for 10 min, and the supernatant was collected and used as enzyme liquid (this operation was done on the ice). Reaction mixture (2.95 mL, 130 mmol/L Met, 750 µmol/L NBT, 100 µmol/L EDTA-Na_2_, 20 µmol/L riboflavin, and 0.05 mol/L pH 7.8 PBS) was taken in a test tube, then enzyme solution (50 μL) was added to it. PBS (50 µL) was used as control group. It was shaken well for mixing and then one tube was placed in the dark and the other tubes were placed for 20 min under light treatment of 4000 Lx. One unit of SOD is defined as the amount of enzyme that inhibited the rate of nitroblue tetrazolium reduction by 50%.

### 3.13. Peroxidase Activity (POD)

POD activity was determined by the 2-methoxyphenol method [45]. Powdery sample (0.3 g) was added into precooled 0.1 mol/L pH 7.0 PBS (3 mL). Then the sample mixture was centrifuged at 4 °C, 12,000 RPM for 10 min, and the supernatant was collected and used as enzyme liquid (this operation was done on ice). Reaction solution (3 mL, 0.2 mol/L pH 6.0 PBS, 2-methoxyphenol, 30% hydrogen peroxide) was taken in a test tube, then enzyme solution (30 µL) was added into it. pH 7.0 PBS (0.1 mol/L) was used as control. Absorbance was taken at 470 nm (3 min). The change of absorbance of 0.01 per min was taken as 1 enzyme activity unit (U).

### 3.14. Catalase Activity (CAT)

CAT activity was determined following the method of Verma et al. [46]. Reaction solution (3 mL, 0.15 mol/L pH 7.0 PBS, 30% hydrogen peroxide) was taken in a test tube, then enzyme solution (0.1 mL) was added to it. Absorbance was determined at 240 nm. pH 7.0 PBS (0.15 mol/L) was used as control. The change of absorbance of 0.1 per min was taken as 1 enzyme activity unit (U).

### 3.15. Statistical Analysis

Results were expressed as mean ± standard (mean ± SD). Each value was the average of three repetitions. A difference between developmental stages of fruit was considered statistically significant (*p* < 0.05) by Tukey’s test. The Pearson correlation analysis was subjected to 2-tailed analysis of variance using the SPSS 17.0.

## 4. Conclusions

In conclusion, this experiment excluded the influence of environment and other factors and proved that the difference in antioxidant enzyme activities in blueberry was mainly due to fruit maturation. Our results showed that phytochemical profiles and antioxidant enzyme activities differed greatly between different developmental stages and tissues. The main antioxidants in green fruit are TPC, TFC and PPO; and main antioxidants during red fruit stage are POD and CAT; main antioxidants at blue fruit stage are TAC, vit. C and SOD. The changes of antioxidant activity during the three key periods of ripening fruit are attributable to the transformation of chemical substances and the interaction of antioxidant enzymes. This study contributes to better understand the influence of developmental blueberry fruit stages on its phytochemical profiles and antioxidant enzyme activity.

## Figures and Tables

**Table 1:** Basic physical properties of blueberry fruit at different developmental stages.

Growth Stages	Transverse Diameter (cm)	Vertical Diameter (cm)	Fruit Shape Index	Fruit Weight (g)	Dry Matter (%)	Water Content (%)
Green	0.97	0.98	1.01	0.43	14.95	85.05
Pink	0.98	0.98	1.00	0.63	14.36	85.64
Red	1.13	1.03	0.91	0.67	13.95	86.05
Purple	1.13	1.13	1.00	0.77	14.29	85.71
Blue	1.29	1.20	0.93	1.11	15.06	84.94
Skin	--	--	--	--	15.19	84.81
Pulp	--	--	--	--	15.15	84.85

**Table 2:** Anthocyanin compounds identified from fruits of blueberry cultivars.

Peak No.	T_R_ (min) ^a^	Identification	Reference ^b^
1	24.92	Delphinidin-3-glucoside	Standard
2	26.17	Delphinidin-3-arabinoside	Grace M H et al. (2009)
3	27.14	Cyanidin-3-galactoside	Grace M H et al. (2009)
4	28.16	Cyanidin-3-arabinoside	Grace M H et al. (2009)
5	29.49	Cyanidin-3-glucoside	Standard
6	30.30	Peonidin-3-galactoside	Grace M H et al. (2009)
7	31.06	Petunidin-3-glucoside	Standard
8	33.73	Peonidin-3-glucoside	Standard
9	34.33	Malvidin-3-galactoside	Grace M H et al. (2009)
10	35.15	Malvidin-3-glucoside	Standard
11	36.30	Malvidin-3-arabinoside	Grace M H et al. (2009)
12	36.65	Delphenidin-6-acetyl-3-glucoside	Grace M H et al. (2009)

^a^ T_R_, retention time. ^b^ References, in the table marked with ‘standard’ mean that they were identified with their corresponding standards. And other mean that they were identified experimentally by reference literatures.

**Table 3:** Correlation between the total phenolic content (TPC), total flavonoid content (TFC), total anthocyanin content (TAC), vitamin C content (vit. C), superoxide dismutase activity (SOD), peroxidase activity (POD), polyphenol oxidase activity (PPO) and catalase activity (CAT).

	TPC	TFC	TAC	vit. C	SOD	POD	PPO	CAT
**TPC**	1.00							
**TFC**	0.91 **	1.00						
**TAC**	0.93 **	0.71 **	1.00					
**vit. C**	0.41	0.09	0.70 **	1.00				
**SOD**	0.63 **	0.63 **	0.65 **	0.50 *	1.00			
**POD**	0.02	0.24 ^a^	0.16	0.17	0.21 ^a^	1.00	-	
**PPO**	0.11 ^a^	0.17	0.34 ^a^	0.54 ^a,^*	0.01 ^a^	0.60 ^a,^**	1.00	
**CAT**	0.72 **	0.53 *	0.78 **	0.55 *	0.44 *	0.42	0.45 ^a,^*	1.00

^a^ showed negative correlation. ** Correlation is significant at the 0.01 level (2-tailed). * Correlation is significant at the 0.05 level (2-tailed).
